# Effects of vitamin D3, omega-3 fatty acids and a simple home exercise program on change in physical activity among generally healthy and active older adults: The 3-year DO-HEALTH trial

**DOI:** 10.1016/j.jnha.2025.100528

**Published:** 2025-03-06

**Authors:** Kariem Hussein, Melanie Kistler-Fischbacher, Michèle Mattle, Caroline De Godoi Rezende Costa Molino, Li-Tang Tsai, Reto W. Kressig, E. John Orav, José A.P. Da Sliva, Bruno Vellas, René Rizzoli, Gabriele Armbrecht, Egli Andreas, Bess Dawson-Hughes, Heike A. Bischoff-Ferrari, DO-HEALTH Research group

**Affiliations:** a Centre on Aging and Mobility, University of Zurich, Zurich, Switzerland; b Department of Geriatric Medicine and Aging Research, University of Zurich, Zurich, Switzerland; c University of Basel, Department of Aging Medicine, Felix Platter, Basel, Switzerland; d Department of Biostatistics, Harvard T.H., Chan School of Public Health, Boston, MA, United States; e Centro Hospitalar e Universitário de Coimbra, Centre for Innovation in Biomedicine and Biotechnology (CIBB), Faculty of Medicine, University of Coimbra, Coimbra, Portugal; f Gérontopôle de Toulouse, Institut du Vieillissement, Centre Hospitalo-Universitaire de Toulouse, UMR INSERM 1027, University of Toulouse III, Toulouse, France; g Division of Bone Diseases, Geneva University Hospitals and Faculty of Medicine, Geneva, Switzerland; h Department of Radiology, Charité-Universitätsmedizin Berlin, Corporate Member of Freie Universität Berlin and Humboldt-Universität Zu Berlin, Berlin, Germany; i Jean Mayer USDA Human Nutrition Research Center on Aging, Tufts University, Boston, Massachusetts, United States

**Keywords:** Dietary supplements, Nutritional supplements, Exercise, Healthy aging, Physical performance, Lifestyle interventions

## Abstract

**Objectives:**

Physical function and physical activity (PA) are key drivers of health and autonomy at older age. We examined the effects of supplemental vitamin D3, supplemental marine omega-3 fatty acids (omega-3s), and a simple home exercise program (SHEP), alone or in combination, on change in physical function and PA among generally healthy older adults.

**Design:**

Multi-center, 2 × 2 × 2 factorial design, randomized controlled trial, follow-up of three years

**Methods:**

Self-reported PA and physical function were pre-defined outcomes of the DO-HEALTH trial, which included older adults (≥70 years) free of major comorbidities. The interventions were vitamin D3 (2000 IU/d), marine omega-3s (1 g/d), and a SHEP (3 × 30 min/wk), applied alone or in combination in eight treatment arms. The outcomes were change in PA (self-reported total PA, metabolic equivalent [MET] h/wk) and physical function (five times sit-to-stand test, hand grip strength, gait speed) from baseline to 12, 24 and 36 months. Mixed effect models were used and adjusted for age, sex, BMI, prior fall, time and baseline level of the outcome.

**Results:**

All 2157 DO-HEALTH participants (mean age 75 years; 83% physically active; 59% vitamin D3 replete) were included. Baseline PA was 75 MET h/wk. Participants receiving omega-3s versus no omega-3s and randomized to SHEP versus control exercise did not differ in PA change over 3 years. However, participants receiving vitamin D3 compared to those receiving no vitamin D3 (Δadjusted means: −7.1 [95% CI −12.7, −1.5] MET h/wk, *P* = 0.01) showed a decline in PA. Results did not differ in subgroups by sex and age (70−74 yrs, ≥75 yrs). Vitamin D3, omega-3s or SHEP did not improve physical function.

**Conclusion:**

Among generally healthy, active, and largely vitamin D3 replete adults aged 70 years and older, vitamin D3, omega-3s and SHEP, individually and in combination had no benefits on self-reported PA and objectively measured physical function. The detrimental effect of vitamin D supplementation on PA change needs further examination.

## Highlights

• High-dose vitamin D3 supplementation (2000 IU/d) may have detrimental effects on physical activity.
• Vitamin D3 supplementation had no beneficial effects on physical function.
• Omega-3s supplementation (1 g/d) had no effect on physical function or physical activity.
• The combination of vitamin D and omega-3s, had negative effects on gait speed.
• The simple home exercise program (SHEP) did not improve physical activity or physical function.

## 1 Introduction

Physical activity (PA) declines with increasing age [[1], [2], [3]] and is associated with disability, risk for falls, risk for fractures, loss of independence and mortality [4]. Conversely, higher levels of PA have been linked to a lower risk of many chronic diseases [5], including cancer [6], dementia [7] and cardio-vascular disease [8] plus a major reduction in premature mortality [9]. Effective strategies to prevent PA decline in older adults, which can be implemented at a public health level, are therefore urgently needed.

PA is defined as any bodily movement produced by skeletal muscles that requires energy expenditure [10]. Physical function is a multidimensional concept, that includes mobility (lower extremity function), dexterity (upper extremity function) and ability to carry out instrumental activities of daily living [11]. In the present manuscript, physical function refers to the performance in different muscle function and strength tests, including gait speed, sit-to-stand test (STS) and hand grip strength.

PA and physical function are interdependent and thus share similar mechanistic pathways. Vitamin D exerts its effect on muscle directly through the vitamin D receptor, and indirectly through the regulation of calcium absorption, and calcium is crucial for muscle contraction [12]. Several observational studies suggest a positive association of higher vitamin D levels and physical function [13,14]; however, results from meta-analyses of randomized controlled trials evaluating the effect of vitamin D supplementation on physical function are conflicting [[15], [16], [17], [18], [19], [20]]. Newer evidence consistently shows no [[19], [20], [21]], or even negative effects [16,18]. In the DO-HEALTH trial, daily vitamin D supplementation (2000 IU) had no benefit on lower extremity function assessed by the Short Physical Performance Battery (SPPB) among generally healthy older adults [22]. In line with those findings, the VITAL trial, which tested the same dose of Vitamin D as DO-HEALTH, reported no benefits for gait speed, grip strength, STS performance or SPPB score among middle-aged to older adults (mean age 67 years) [23].

Omega-3 fatty acids (Omega-3s) may improve muscle function through anti-inflammatory pathways, and increased protein synthesis [24,25]. However, studies on the effect of omega-3s supplementation on physical function among older adults are sparse. Nevertheless, a small meta-analysis of RCTs suggests that omega-3s supplementation improves timed up-and-go test time (4 trials), and gait speed (2 trials) among adults aged 60 years and older [26], while no benefits were found for grip and leg strength. Also the VITAL trial reported no benefit of daily supplementation with omega-3s on physical function [23].

Exercise provides anabolic (muscle protein synthesis) and neural (muscle fiber recruitment) stimuli and is the optimal strategy to improve physical function [27]. Indeed, meta-analyses of RCTs consistently report a positive effect of exercise on physical performance in older adults [[28], [29], [30]].

While the effects of vitamin D, omega-3s and exercise on physical function have been examined, it is largely unknown if, and to what extent, the three interventions can improve PA. Therefore, the aim of the present study was to examine the effect of daily supplemental vitamin D3, daily supplemental omega-3s and a simple home exercise program (SHEP), alone or in combination, on PA and physical function in generally healthy older adults living in the community.

## 2 Methods

### 2.1 Study design

The Vitamin D3-, Omega3- and Home Exercise- Healthy Ageing and Longevity Trial (DO-HEALTH) was a three-year, multi-center, double-blind, randomized, placebo-controlled, 2 × 2 × 2 factorial design trial, including 2157 generally healthy older adults from 5 European countries (Switzerland, Germany, Austria, France, Portugal). DO-HEALTH examined the individual and combined effects of vitamin D3, omega-3 fatty acids and SHEP on six primary outcomes (change in systolic and diastolic blood pressure, Short Physical Performance Battery [SPPB], Montreal Cognitive Assessment score, and incidence rates of non-vertebral fractures and infections) [22]. Self-reported PA was a pre-defined exploratory outcome of DO-HEALTH [31]. SPPB was a primary outcome as reported previously [22]. We report individual components of the SPPB, including gait speed and STS, as well as hand grip strength as secondary outcomes in this analysis.

The study protocol was approved by the ethics commissions of all five countries and registered in the International Trials Registry (clinicaltrials.gov, registration ID: NCT01745263) [31]. STS and hand grip strength were pre-defined secondary outcomes of DO-HEALTH. Gait speed was added as an additional outcome due to its high relevance for monitoring functional status and overall health [32]. The approval for gait speed analyses was obtained after trial completion from the ethics committee in Zurich (2024-02091). All participants gave written informed consent and all study procedures were conducted in accordance with the Declaration of Helsinki.

### 2.2 Participants

Participants were recruited from seven study centers (Zurich, Basel, Geneva, Berlin, Innsbruck, Toulouse, Coimbra). The full list of eligibility criteria has been published [31]. In brief, participants had to be 70 years or older, living independently in the community, sufficiently mobile to come to the study center, able to walk 10 meters without assistance and ablet to get in and out of a chair without help. Furthermore, they had to have good cognitive function, defined as a score of ≥24 points on the Mini Mental State Examination. Exclusion criteria relevant to the outcomes reported in the present analysis included a history of major health events (i.e., myocardial infarction, cancer) in the five years prior to enrollment, hemiplegia, severe gait impairment and more than three falls in the month prior to enrollment. Furthermore, participants had to be willing to limit vitamin D supplementation to a daily maximum of 800 IU and to refrain from omega-3s supplementation for the duration of the trial.

### 2.3 Study interventions

The three interventions were 2000 IU/d of vitamin D3 versus placebo, 1 g/d of omega-3s versus placebo and a simple home exercise program (SHEP) versus a control exercise program. Each participant received 2 capsules per day. Verum vitamin D3 capsules contained 1000 IU of vitamin D3, stabilized with dl-α-tocopherol (vitamin E, 2.5 pro mill). Verum omega-3s capsules contained 500 mg of eicosapentaenoic acid (EPA) and docosahexaenoic acid (DHA) in a ratio of 1:2. Placebo capsules contained high oleic sunflower oil.

The SHEP consisted of the following five exercises: sit-to-stand, single-leg stance, pull-back against elastic resistance, external shoulder rotation against elastic resistance, and stepping up and down one step on stairs. Three sets of 10 repetitions were performed for each exercise, except for single-leg stance, for which 10 sets of 10 sec were performed for each leg. The control program was expected to have no benefit on the endpoints tested in DO-HEALTH and consisted of five exercises aiming to improve hip, knee, ankle, spine, and shoulder mobility. All participants were instructed to complete the exercise program three times per week. A paper booklet with detailed instructions and an animated motivational video of the exercise program were provided to the participants. During the baseline visit, the program was instructed by a physiotherapist who was not involved in the assessment of the trial outcome measures. The physiotherapist also instructed the participant on the training strategy (e.g., reduce the number of repetitions or taking a one-week break if there was pain associated with the exercises). If there were any problems with the exercise program during the follow-up, participants were asked to call the recruitment center and ask for a call back from the physiotherapist, without mentioning the issue or the program. If participants wanted to increase training volume, they were invited to repeat the full program [31].

Adherence to the study interventions was assessed at each 3-monthly contact (phone calls and clinical visits). Participants were given a study diary as a support tool to record and report adherence to all three interventions to the study staff. In addition to self-reported data, adherence to vitamin D3 and omega-3s supplements was assessed by measuring 25(OH)D and polyunsaturated fatty acid blood levels and through capsule counts of used, partially used and full bottles of study capsules, returned by the participants at each visit.

### 2.4 Allocation and masking

Allocation to the eight treatment arms was computer-based (DO-HEALTH randomization software) and performed using block randomization (block size of 16 individuals) stratified by sex, age (70–84 years or ≥85 years), study center, history of falling in the 12 months prior to enrolment (yes/no). All study participants, staff and investigators were blinded to treatment allocation, except for a physiotherapist who instructed the exercise programs but was not involved in the data collection. All assessments and examinations were performed by trained study staff, according to standard operating procedures.

### 2.5 Outcomes

PA was self-reported by participants and assessed with an excerpt of the Nurses’ Health Study Physical Activity Questionnaire (NHS-PAQ) [33]. The NHS-PAQ assesses average time spent with different recreational activities per week (e.g., walking, cycling, tennis, swimming, resistance training). Participants were asked to indicate how many minutes or hours per week they spend with each activity. Based on the reported times and energy expenditure measured in metabolic equivalents (MET) for each activity, the total MET-hours/week were calculated. Details on the coding of the NHS-PAQ are published elsewhere [2]. In brief, the intensity of each activity reported in the NHS-PAQ was classified as light (<3 METs), moderate (3–6 METs) and vigorous (≥6 METs), following the guidelines outlined in the physical activity compendium [34]. To account for over-reporting but avoid capping of too many datapoints, we calculated the time per week spent with light, moderate and vigorous activities and capped the sum of light PA at 24 h/day, moderate PA at 35 h/week and vigorous PA at 21 h/week. The PA outcome reported here is defined as the sum of light, moderate and vigorous activities.

Objective measures of physical function included five times sit-to-stand (STS) test, hand grip strength of the dominant hand and gait speed. Gait speed and STS were measured as components of the SPPB. The time (seconds) to complete the STS test was measured from initial sitting position to the final (fifth repetition) standing position. Gait speed (m/s) was measured over a distance of four meters [35]. Hand grip strength was measured with a Martin Vigorimeter (KLS Martin KG, Tuttlingen, Germany) and the best out of three consecutive attempts was used [36].

### 2.6 Statistical analyses

Baseline clinical and demographic characteristics are described overall and by treatment groups. Normally distributed continuous variables are presented as mean and standard deviation (SD) and non-normally distributed variables as median and interquartile range (IQR). Categorical variables are presented in frequencies and percentages.

Change in self-reported PA, STS, gait speed, and hand grip strength from baseline to year 1, 2 and 3 was analyzed using separate mixed effects models with an unstructured dependence structure. Randomization stratification factors including age, linear spline at age 85, sex, prior fall, and study site were adjusted in these models as covariates along with time, BMI, and corresponding baseline level of the outcome. To determine whether the treatment effects are additive, both three-way and three two-way interaction effects were examined for all reported outcomes. If none of the treatment interaction effects were significant (p < 0.05), three dichotomous indicators for vitamin D, omega-3, and SHEP were added to the model to examine additive treatment effects. Otherwise, an eight-level treatment group categorical variable was added to the model to examine non-additive treatment effects. Effects of treatment, time, and treatment by time interaction were examined in the mixed effects models. Adjusted means and 95% confidence intervals are reported.

Subgroup analyses were performed by sex (male, female) and age (70−74 yrs, ≥75 yrs). Subgroup analysis first assessed the significance of the interaction between subgroup and treatment. If the interaction was significant (p < 0.05), stratified analyses were performed by each level of the subgroup factor.

Additionally, the association between quartiles of achieved 25(OH)D levels and change in PA over three years was calculated in a post-hoc analysis. The exposure of quartiles of achieved 25(OH)D levels was calculated based on the mean achieved 25(OH)D levels across years 1, 2, and 3. Mixed effects models with an unstructured dependence structure controlled for age, linear spline at age 85, sex, prior fall, study site, BMI, time, baseline PA, and treatment effects of omega-3s and SHEP were used to test the association. The model did not include the treatment effect of vitamin D3, as the latter may be considered on the pathway of the association.

All analyses were performed in SAS v9.4 statistical software (Copyright© 2004 by SAS Institute Inc., Cary, NC, USA) and R Studio. Significance level was set at 0.05 (two-sided).

Power calculations were based on primary outcomes [31] and therefore no sample size calculations were conducted for the secondary and exploratory outcomes reported in the present analysis.

## 3 Results

### 3.1 Study participants

A total of 2157 participants were included. The mean follow-up time was 2.99 years and 88% of participants completed the trial. The CONSORT diagram of participant flow has previously been published [22]. Participant characteristics at baseline are presented in Table 1. The mean age of participants was 74.9 years and 61.7% were women. Participants reported a mean Sangha comorbidity score of 3.3 and 42% reported at least one fall in the 12 months prior to enrollment. Mean gait speed was 1.2 m/s, median SPPB score was 11 (of a maximum of 12) points and only 2% used a walking aid. Most participants (83%) reported to engage in some type of PA at least once a week. At baseline, mean 25(OH)D serum concentration was 22.4 ng/mL and 40.7% of participants were vitamin D deficient based on a cutoff of 20 ng/mL. Detailed data on adherence have been published elsewhere [31]. In brief, based on participant self-report, 86% of participants took at least 80% of their total study capsules and 70% performed the exercise program at least twice per week over the 3-year follow-up (Supplemental Table S1). At year 3, participants who received vitamin D3 had higher mean serum concentrations of 25(OH)D compared to those receiving placebo (37.6 vs 24.4 ng/mL). Similarly, participants who received omega-3s had higher concentrations of DHA and EPA compared to those receiving placebo (135.6 vs 76.3 μg/mL for DHA and 64.7 vs 33.8 μg/mL for EPA at 3 years; eTable 5 and eFig. S1 in Supplement 2 of Bischoff-Ferrari et al. 2020 [22])

**Table 1:** Baseline characteristics of study participants.

		Vitamin D3		Omega-3s		Exercise	
Characteristics^a^	Overall (n = 2157)	Vitamin D3 (n = 1076)	No vitamin D3 (n = 1081)	Omega-3s (n = 1073)	No omega-3s (n = 1084)	SHEP (n = 1081)	Control exercise (n = 1076)
Age [yrs], mean (SD)	74.9 (4.4)	75.0 (4.5)	74.9 (4.4)	74.7 (4.3)	75.2 (4.6)	75.0 (4.5)	74.9 (4.4)
Age categories, n (%) [yrs]							
70−74	1237 (57.3)	606 (56.3)	631 (58.4)	635 (59.2)	602 (55.5)	622 (57.5)	615 (57.2)
75+	920 (42.7)	470 (43.7)	450 (41.6)	438 (40.8)	482 (44.5)	459 (42.5)	461 (42.8)
BMI [kg/m^2^], mean (SD)^b^	26.3 (4.3)	26.5 (4.4)	26.2 (4.2)	26.3 (4.2)	26.4 (4.3)	26.3 (4.2)	26.4 (4.4)
Sex, n (%)							
Women	1331 (61.7)	667 (62.0)	664 (61.4)	668 (62.3)	663 (61.2)	665 (61.5)	666 (61.9)
Men	826 (38.3)	409 (38.0)	417 (38.6)	405 (37.7)	421 (38.8)	416 (38.5)	410 (38.1)
Comorbidity score, mean (SD)^c^	3.3 (3.0)	3.3 (3.1)	3.3 (3.0)	3.3 (3.1)	3.3 (2.9)	3.2 (3.0)	3.4 (3.1)
Prior fall, n (%)	903 (41.9)	446 (41.5)	457 (42.3)	441 (41.1)	462 (42.6)	450 (41.6)	453 (42.1)
Use of walking aid, n (%)	41 (1.9)	21 (2.0)	20 (1.9)	26 (2.5)	15 (1.4)	26 (2.4)	15 (1.4)
Total physical activity [MET h/wk], median (IQR)	74.9 (40.8, 138.2)	73.0 (39.1, 138.1)	75.6 (42.4, 138.7)	76.7 (42.3, 138.3)	73.2 (39.4, 138.0)	75.4 (40.1, 139.9)	73.5 (41.4, 135.8)
Physical activity frequency							
None, n (%)	375 (17.4)	207 (19.2)	168 (15.5)	190 (17.7)	185 (17.1)	179 (16.6)	196 (18.2)
1−2 times per week, n (%)	652 (30.2)	318 (29.6)	334 (30.9)	311 (29.0)	341 (31.5)	323 (29.9)	329 (30.6)
≥ 3 times per week, n (%)	1128 (52.3)	550 (51.1)	578 (53.5)	570 (53.1)	558 (51.5)	578 (53.5)	550 (51.1)
4-m gait speed [m/s], mean (SD)	1.2 (0.2)	1.2 (0.2)	1.2 (0.2)	1.2 (0.2)	1.2 (0.2)	1.2 (0.2)	1.2 (0.2)
STS time [s], median (IQR)	10.7 (8.8, 13.5)	10.6 (8.8, 13.4)	10.9 (8.8, 13.5)	10.6 (88, 13.4)	10.8 (8.8, 13.6)	10.7 (8.9, 13.6)	10.8 (8.7, 13.4)
Grip strength [kPa], mean (SD)	60.2 (18.6)	60.1 (18.8)	60.2 (18.4)	59.8 (18.4)	60.6 (18.7)	59.9 (18.2)	60.5 (18.9)
SPPB score, median (IQR)^d^	11 (10, 12)	12 (10, 12)	11 (10, 12)	11 (10, 12)	11 (10, 12)	11 (10, 12)	11 (10, 12)
Serum 25(OH)D concentration [ng/mL], mean (SD)	22.4 (8.4)	22.4 (8.4)	22.4 (8.5)	22.4 (8.4)	22.4 (8.4)	22.8 (8.6)	22.0 (8.3)
Vitamin D3 deficiency (< 20 ng/mL), n (%)	872 (40.7)	427 (40.1)	445 (41.4)	422 (39.7)	450 (41.8)	422 (39.4)	450 (42.1)
Serum DHA concentration [μg/mL], mean (SD)	78.1 (36.9)	78.1 (37.9)	78.1 (35.9)	78.9 (37.2)	77.3 (36.6)	78.2 (36.5)	78.0 (37.4)
Serum EPA concentration [μg/mL], median (IQR)	25.5 (18.1, 37.7)	24.8 (17.4, 37.7)	26.2 (18.6, 37.7)	26.1 (18.5, 37.7)	25.3 (17.6, 37.9)	25.1 (17.5, 37.6)	25.9 (18.6, 38.1)

Abbreviations: BMI, body mass index; DHA, docosahexaenoic acid; EPA, eicosapentaenoic acid; IQR, interquartile range; MET, metabolic equivalent; SD, standard deviation; SHEP, simple home exercise program; SPPB, short physical performance battery; STS, five times sit-to-stand; wk, week; yrs, years.

a Median and IQR are presented for non-normally distributed variables.

b Body mass index (BMI) was calculated as weight in kilograms divided by height in meters squared.

c Comorbidity was measured by the Self-Administered Comorbidity Questionnaire, which assesses 12 comorbidities by 3 dimensions (presence, medication, and limitation of activities). It has a range of 0–36 points and lower scores indicate better health.

d The Short Physical Performance Battery (SPPB) assesses lower extremity function. Scores range from 0 to 12, in which higher scores are better.

### 3.2 Self-reported physical activity

**Fig. 1:**
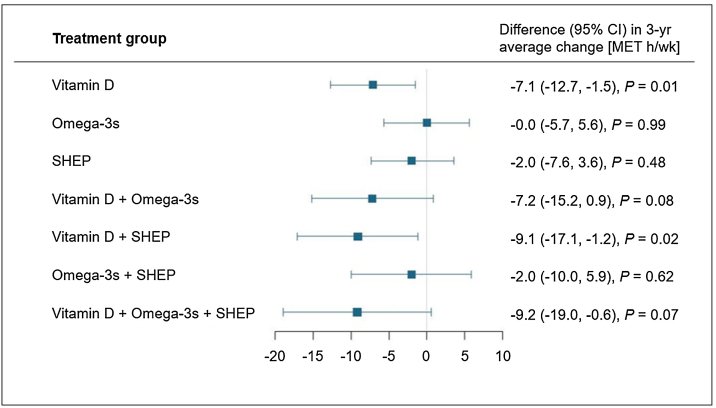
3-year average change in physical activity for treatment combinations versus the respective comparison groups not receiving the treatment. Abbreviations: MET, metabolic equivalent; SHEP, simple home exercise program. Analyses were adjusted for age, linear spline at age 85, sex, prior fall, study site, BMI, time and baseline PA.

For self-reported PA there were no treatment interactions. Therefore, main effects are presented and treatment effects are additive. Omega-3s and SHEP had no influence on self-reported PA change across the 3-year follow-up. However, participants receiving vitamin D3 compared to participants receiving no vitamin D3 showed a greater decline in self-reported PA (Δ adjusted means: −7.1 [95% CI −12.7, −1.5] MET h/wk, *P* = 0.01) (Table 2). Also, the combination of vitamin D3 and SHEP resulted in a decline in self-reported PA over 3 years (Δ adjusted means: −9.7 [95% CI −17.1, −1.2] MET h/wk, *P* = 0.02; Fig. 1 and Supplemental Table S4). There were no significant interactions between any of the subgroups and treatments for PA (Supplemental Table S2).

**Table 2:** Change from baseline in self-reported PA by treatment group.

Total PA [MET h/wk]^a^	Vitamin D3	No vitamin D3	Difference in adjusted means	*P* value	Omega-3s	No Omega-3s	Difference in adjusted means	*P* value	SHEP	Control exercise	Difference in adjusted means	*P* value
**Total PA** [MET h/wk]												
Unadjusted at baseline (n = 2155)	101.3 (96.0, 106.7)	103.4 (98.2, 108.7)	2.1 (−5.4, 9.5)	0.59	105.2 (99.8, 110.7)	99.6 (94.6, 104.6)	−5.6(− 13.1, 1.9)	0.14	102.7 (97.4, 107.9)	102.1 (96.7, 107.5)	−0.6(− 8.1, 6.9)	0.88
Adjusted change from baseline												
Year 1	−3.7 (−9.2, 1.9)	5.0(−0.5, 10.5)	−8.6 (−16.4, −0.9)		0.7(−4.9, 6.2)	0.6(−4.8, 6.1)	0.1 (−7.7, 7.8)		−0.3(−5.8, 5.2)	1.6(−3.9, 7.1)	−1.9(−9.7, 5.8)	
Year 2	−8.3 (−13.7, −2.8)	0.2(−5.2, 5.7)	−8.5 (−16.2, −0.8)		−3.2(−8.7, 2.2)	−4.8(−10.2, 0.6)	1.5 (−6.2, 9.2)		−5.4(−10.8, 0.0)	−2.6(−8.1, 2.8)	−2.7(−10.4, 5.0)	
Year 3	−11.0 (−16.5, −5.5)	−6.8(−12.4, −1.3)	−4.2 (−12.0, 3.7)		−9.8(−15.3, −4.2)	−8.1(−13.5, −2.6)	−1.7 (−9.5, 6.1)		−9.6(−15.1, −4.1)	−8.2(−13.7, −2.7)	−1.4(−9.2, 6.4)	
Average across 3 years	−7.6 (−11.6, −3.7)	−0.5(−4.5, 3.4)	−7.1 (−12.7, −1.5)	0.01	−4.1(−8.1, −0.1)	−4.1(−8.0, −0.1)	−0.0(−5.7, 5.6)	0.99	−5.1(−9.1, −1.1)	−3.1(−7.1, 0.9)	−2.0(−7.6, 3.6)	0.48

Abbreviations: CI, confidence interval; MET, metabolic equivalent; PA, physical activity SHEP, simple home exercise program.

a Treatment effects are derived from mixed effects models with change from baseline as the outcome. No significant interaction between treatment groups and time was observed, so the model omitted the interaction term for treatment*time. The model controls age, linear spline at age 85, sex, body mass index, prior fall, study site, time and baseline PA.

### 3.3 Objective measures of physical function

For grip strength there was a significant three-way treatment interaction (*P* = 0.004). Consequently, the seven treatment combinations were compared to placebo. There were no significant effects of any of the treatments on grip strength (Table 3). There were no significant interactions between any of the subgroups and treatments for grip strength (Supplemental Table S3).

**Table 3:** Change in gait speed and dominant hand grip strength from baseline by treatment group.

Differences in adjusted means compared to placebo (95% CI)^a^					
Treatment group	Unadjusted at baseline	Year 1	Year 2	Year 3	3-year average change
Gait speed [m/s]	n = 2153				
Vitamin D3	1.16 (1.13, 1.19)	0.01 (−0.02, 0.04)	0.01 (−0.02, 0.04)	−0.00 (−0.03, 0.03)	0.00 (−0.02, 0.03) *P* = 0.66
Omega-3s	1.18 (1.15, 1.21)	0.01 (−0.02, 0.04)	0.00 (−0.03, 0.03)	−0.01 (−0.04, 0.02)	−0.00 (−0.02, 0.02), *P* = 0.99
SHEP	1.18 1.14, 1.19)	−0.01 (−0.04, 0.02)	0.00 (−0.03, 0.03)	−0.03 (−0.06, 0.00)	−0.01 (−0.03, 0.01), *P* = 0.32
Omega-3s + vitamin D3	1.16 (1.13, 1.19)	−0.02 (−0.05, 0.01)	−0.02 (−0.05, 0.01)	−0.03 (−0.06, −0.00)	−0.03 (−0.05, −0.00), *P* = 0.03
Vitamin D3 + SHEP	1.16 (1.13, 1.18)	0.01 (−0.01, 0.04)	0.01 (−0.02, 0.04)	0.00 (−0.03, 0.03)	0.01 (−0.01, 0.03), *P* = 0.45
Omega-3s + SHEP	1.16 (1.13, 1.19)	−0.01 (−0.03, 0.02)	0.01 (−0.02, 0.04)	−0.00 (−0.03, 0.03)	−0.00 (−0.02, 0.02), *P* = 0.99
Vitamin D3 + omega-3s + SHEP	1.17 (1.14, 1.20)	−0.02 (−0.04, 0.01)	−0.01 (−0.04, 0.02)	−0.01 (−0.04, 0.02)	−0.01 (−0.04, 0.01), *P* = 0.32
Grip strength [kPa]	n = 2152				
Vitamin D3	60.82 (58.63, 63.02)	0.68 (0.67, 2.02)	1.38 (0.26, 3.02)	0.91 (−1.03, 2.86)	0.87 (−0.28, 2.02), *P* = 0.14
Omega-3s	59.70 (57.51, 61.89)	0.17 (−1.19, 1.52)	1.50 (−0.15, 3.15)	1.01 (−0.93, 2.96)	0.62 (−0.54, 1.78), *P* = 0.29
SHEP	59.49 (57.35, 61.62)	0.42 (−0.94, 1.78)	1.38 (−0.27, 3.02)	0.68 (−1.28, 2.65)	0.67 (−0.49, 1.84), *P* = 0.26
Omega-3s + vitamin D3	59.64 (57.27, 62.00)	−0.64 (−2.00, 0.73)	−0.71 (−2.36, 0.94)	−1.03 (−2.99, 0.93)	−0.74 (−1.9, 0.43), *P* = 0.22
Vitamin D3 + SHEP	60.22 (57.90, 62.53)	−0.58 (−1.92, 0.76)	−0.43 (−2.05, 1.18)	−0.65 (−2.56, 1.26)	−0.57 (−1.71, 0.58), *P* = 0.33
Omega-3s + SHEP	60.07 (57.92, 62.22)	0.49 (−0.86, 1.84)	0.91 (−0.73, 2.54)	−0.06 (−2.0, 1.89)	0.45 (−0.70, 1.61), *P* = 0.44
Vitamin D3 + omega-3s + SHEP	59.81 (57.66, 61.96)	−0.35 (−1.03, 1.72)	0.29 (−1.37, 1.94)	0.64 (−1.33, 2.62)	0.40 (−0.77, 1.57), *P* = 0.51

Abbreviations: CI, confidence interval; SHEP, simple home exercise program.

a Treatment effects are derived from mixed effects models with change from baseline as the outcome. No significant interaction between treatment groups and time was observed for either outcome, so the model omitted the interaction term for treatment*time. However, significant treatment group interactions were observed for both outcomes: a three-way treatment interaction (P = 0.004) for grip strength, and a two-way treatment interaction (P = 0.01) for gait speed. Therefore, an eight-level treatment group categorical variable was included in the models for both outcomes. The models control for age, linear spline at age 85, sex, body mass index, prior fall, study site, time and corresponding baseline measure.

For gait speed there was a significant two-way treatment interaction (*P* = 0.01). Consequently, the seven individual treatment arms were compared to placebo. There were no significant effects of any of the treatments except for the vitamin D3 plus omega-3s group. Individuals receiving vitamin D3 plus omega-3s compared with placebo showed a small worsening of gait speed over time (Δ adjusted means: −0.03 [−0.05, −0.00] m/s; *P* = 0.03, Table 3). There were no significant interactions between any of the subgroups and treatments for gait speed (Supplemental Table S1).

For STS there were no significant treatment interactions. Therefore, main effects are presented and treatment effects are additive. Vitamin D3, omega-3s and SHEP had no effect on STS change across the 3-year follow-up (Table 4). Similarly, there were no significant effects of treatment combinations across the 3 years (Supplemental Table S4). There were no significant interactions between any of the subgroups and treatments for STS (Supplemental Table S2).

**Table 4:** Change from baseline in five time sit-to-stand by treatment group.

STS [s] a	Vitamin D3	No vitamin D3	Difference in adjusted mean a	*P* value	Omega-3s	No Omega-3s	Difference in adjusted mean a	*P* value	SHEP	Control exercise	Difference in adjusted mean a	*P* value
Unadjusted at baseline (n = 2131)	11.74 (11.5, 12.0)	11.64 (11.38, 11.89)	0.11 (−0.26, 0.47)	0.57	11.66 (11.39, 11.92)	11.72 (11.47, 11.97)	0.07 (−0.30, 0.43)	0.72	11.82 (11.55, 12.09)	11.56 (11.32, 11.80)	−0.26 (− 0.62, 0.10)	0.16
Adjusted change from baseline												
Year 1	−0.01(−0.19, 0.16)	−0.15(−0.33, 0.02)	0.14(−0.11, 0.39)		0.05 (−0.13, 0.22)	−0.22(−0.39, −0.05)	0.27 (0.02, 0.51)		−0.16 (−0.34, 0.01)	−0.00(−0.18, 0.17)	−0.16(−0.41, 0.09)	
Year 2	−0.22(−0.40, −0.04)	−0.09(−0.27, 0.09)	−0.13(−0.39, 0.13)		−0.14(−0.32, 0.04)	−0.17(−0.35, 0.01)	0.03(−0.22, 0.29)		−0.19(−0.37, −0.01)	−0.12(−0.30, 0.06)	−0.07(−0.33, 0.19)	
Year 3	−0.12(−0.34, 0.09)	−0.07(−0.29, 0.15)	−0.05(−0.36, 0.25)		−0.21(−0.43, 0.01)	0.02(−0.20, 0.23)	−0.23(−0.53, 0.08)		−0.19(−0.41, 0.02)	0.00(−0.22, 0.22)	−0.20(−0.50, 0.11)	
Average across 3 years	−0.12 (−0.27, 0.03)	−0.11 (−0.26, 0.04)	−0.01(−0.23, 0.20)	0.90	−0.10(−0.25, 0.05)	−0.12(−0.27, 0.02)	0.02(−0.19, 0.24)	0.82	−0.18(−0.33, −0.03)	−0.04(−0.19, 0.11)	−0.14(−0.36, 0.07)	0.19

Abbreviations: CI, confidence interval; SHEP, simple home exercise program; STS, five times sit-to-stand.

a Treatment effects are derived from mixed effects models with change from baseline as the outcome. A significant interaction between omega-3s and time was observed (*P* value = 0.009), so the model included the interaction term for treatment*time. Additionally, the model controls for age, linear spline at age 85, sex, BMI, prior fall, study site, time and baseline STS.

### 3.4 Post-hoc analysis achieved 25(OH)D levels and change in PA

Quartiles of mean achieved 25(OH)D levels across year 1, 2, and 3 are presented in Supplemental Table S5. There was a significant difference in mean change from baseline in PA across quartiles of mean achieved 25(OH)D levels over 3 years (Supplemental Table S6). Over the 3-year follow-up, there were significant differences in change from baseline in PA when comparing quartile 3 to 2 (mean difference −9.93 [95% CI −17.97, −1.89] MET h/wk, *P* = 0.02), and quartile 4 to 2 (mean difference −9.81 [95% CI −17.93, −1.69 MET h/wk], *P* = 0.02; Supplemental Table S6). Additionally, no significant interaction was observed between quartiles of mean achieved 25(OH)D levels and treatment with vitamin D3 (*P* = 0.73).

## 4 Discussion

In this multi-center randomized controlled trial of 2157 generally healthy, active and largely vitamin D replete adults age 70 years and older, vitamin D3 supplementation, omega-3s supplementation and a simple home exercise program, applied individually or in combination, did not improve self-reported PA. Also, objective measures of physical function did not improve with any treatments. Moreover, we cannot exclude a detrimental effect of vitamin D alone on self-reported PA and in combination with omega-3s on gait speed.

The lack of benefit of vitamin D, omega-3s and the SHEP on self-reported PA and the three measures of objective physical function (STS, gait speed, grip strength) is consistent with the primary outcome lower extremity function in DO-HEALTH assessed by the SPPB [22]. Also, our finding is in line with the VITAL trial [23] for vitamin D and omega-3s regarding several of the same functional measures.

For omega-3s, a recent meta-analysis among older adults reported mixed findings with no benefits of supplementation for gait speed, grip strength or leg muscle strength, but a significant improvement on the timed up-and-go test [26]. However, the findings of this meta-analysis should be interpreted with caution as it was limited to four studies with a pooled sample size of only 136 participants [26].

The lack of benefit of the SHEP on self-reported PA and objective physical function may be explained by the fact that 83% of DO-HEALTH participants were engaging in PA at least once a week at baseline. Further, the SHEP was of relatively low intensity and unsupervised. Regarding the latter, a recent meta-analysis of 34 RCTs including older adults (≥60 years) suggests that unsupervised exercise may be less effective in improving physical function [29].

Regarding the observed detrimental effect of vitamin D on self-reported PA in DO-HEALTH, recent studies suggest that high dose (>2800 IU) vitamin D may have detrimental effects on falls [37], muscle health and physical function [18,21,37]. The post-hoc analyses on achieved 25(OH)D levels support evidence from these studies and show a detrimental effect for PA only among participants with higher achieved 25(OH)D levels. Thus, we cannot exclude that this negative effect of higher doses of vitamin D may be real. An explanation for this may be that high doses of vitamin D, even if applied daily, may trigger countervailing factors that affect muscle health negatively [38]. In DO-HEALTH 59% of participants were vitamin D replete at baseline and, according to current guidelines, participants were allowed to take vitamin D supplements of up to 800 IU per day, independent of the study intervention, which was implemented by 33% of participants at year 3. By taking the daily dose of 2000 IU vitamin D tested in DO-HEALTH plus the allowed additional intake of 800 IU per day, some participants may have achieved a high enough intake to trigger countervailing factors such as an increase in fibroblast growth factor 23 (FGF-23), which may contribute to a decrease in muscle health [39,40]. Alternatively, a neutral effect of vitamin D on physical function in generally healthy and active older adults without vitamin D deficiency is supported by several recent meta-analyses [[18], [19], [20]] and findings from the VITAL trial [23].

Notably, the detrimental effect of vitamin D supplementation on self-reported PA in DO-HEALTH with 2000 IU vitamin D, does not challenge the recommended daily intake of 800–1000 IU for older adults with vitamin D deficiency, at high fall or fracture risk [41,42]. Similarly, the lack of benefit of the SHEP on self-reported PA in DO-HEALTH does not invalidate the benefits of exercise for muscle health and function in older adults [43].

The large sample size of 2157 participants and the 3-year trial phase are two major strengths of the DO-HEALTH trial, as is its 2 × 2 × 2 factorial design to test individual and additive benefits of the interventions, and the fact that DO-HEALTH was designed as a prevention trial targeting generally healthy adults age 70 and older. However, there are also limitations that should be acknowledged. First, our outcome PA was self-assessed and may be subject to recall bias as well as over- and underreporting. However, our objective measures of physical function are in line with the absence of a benefit on self-reported PA. Second, our restriction of recruitment to generally healthy older adults, limits generalizability of our findings to frailer populations and those at greater risk for vitamin D deficiency and functional decline.

In conclusion, among generally healthy and active older adults, vitamin D3 supplementation, omega-3s supplementation and a simple home exercise program showed no benefits on self-reported physical activity and objectively measured physical function. The observed reduction in self-reported physical activity with daily 2000 IU vitamin D3 supplementation needs further examination.

## DO-HEALTH research group

The members of DO-HEALTH Research Group are Bischoff-Ferrari Heike A, Egli Andreas, Rival Sandrine, Wanner Guido A, Vellas Bruno, Guyonnet Sophie, Rizzoli René, Biver Emmanuel, Merminod Fanny, Kressig Reto W, Bridenbaugh Stephanie, Suhm Norbert, Da Silva José AP, Duarte Cátia CM, Pinto Filipa Ana, Felsenberg Dieter, Börst Hendrikje, Armbrecht Gabriele, Blauth Michael, Spicher Anna, Felson David T, Kanis John A, Mccloskey Eugene V, Johansson Elena, Watzl Bernhard, Gomez Rodriguez Manuel, Hofbauer Lorenz, Tsourdi Elena, Rauner Martina, Siebert Uwe, Kanis John A, Halbout Philippe, Ferrari Stephen M, Gut Benno, Ba Marième, Wittwer Schegg Jonas, Etheve Stéphane, Eggersdorfer Manfred, Delannoy Carla Sofa, Reuschling Monika, Orav Endel J, Willett Walter C, Manson JoAnn E, Dawson-Hughes Bess, Staehelin Hannes B, Walter Paul W, Dick Walter, Fried Michael, von Eckardstein Arnold, Theiler Robert, Simmen Hans-Peter, Langhans Wolfgang, Zinkernagel Annelies, Mueller Nicolas, Distler Oliver, Graetz Klaus, Nitschke Ina, Dietrich Thomas, Baer Walter, Landau Klara, Ruschitzka Frank, Manz Markus, Burckhardt Peter.

## Declaration of competing interest

HAB-F reports as the PI of the DO-HEALTH trial, grants from the 10.13039/501100000780European Commission (Grant Agreement No. 278588), from the University of Zurich, from NESTEC, from PFIZER Consumer Healthcare, from Streuli Pharma, plus non-financial support from DSM Nutritional Products and from Roche Diagnostics. Furthermore, HAB-F reports speaker fees from Wild, Pfizer, Vifor, Mylan, Roche Diagnostics, and independent and investigator initiated grants from 10.13039/100004319Pfizer and from Vifor, outside the submitted work. KH, MK-F, MM, CG, LT, RWK, EJO, JAPS, BV, RR, GA, AE, BD-H declare no conflicts of interest.

## Footnotes

Appendix A Supplementary material related to this article can be found, in the online version, at doi:https://doi.org/10.1016/j.jnha.2025.100528.

## References

[1] Siparsky P.N., Kirkendall D.T., Garrett W.E. (2014). Muscle changes in aging: understanding sarcopenia. Sports Health.

[2] Mattle M., Meyer U., Lang W., Mantegazza N., Gagesch M., Mansky R. (2022). Prevalence of physical activity and sedentary behavior patterns in generally healthy european adults aged 70 years and older-baseline results from the DO-HEALTH clinical Trial. Front Public Health.

[3] Wilkinson D.J., Piasecki M., Atherton P.J. (2018). The age-related loss of skeletal muscle mass and function: measurement and physiology of muscle fibre atrophy and muscle fibre loss in humans. Ageing Res Rev.

[4] Cunningham C., O’Sullivan R., Caserotti P., Tully M.A. (2020). Consequences of physical inactivity in older adults: a systematic review of reviews and meta-analyses. Scand J Med Sci Sports.

[5] Anderson E., Durstine J.L. (2019). Physical activity, exercise, and chronic diseases: a brief review. Sports Med Health Sci.

[6] Jurdana M. (2021). Physical activity and cancer risk. Actual knowledge and possible biological mechanisms. Radiol Oncol.

[7] Iso-Markku P., Kujala U.M., Knittle K., Polet J., Vuoksimaa E., Waller K. (2022). Physical activity as a protective factor for dementia and Alzheimer’s disease: systematic review, meta-analysis and quality assessment of cohort and case-control studies. Br J Sports Med.

[8] Isath A., Koziol K.J., Martinez M.W., Garber C.E., Martinez M.N., Emery M.S. (2023). Exercise and cardiovascular health: a state-of-the-art review. Prog Cardiovasc Dis.

[9] Saint-Maurice P.F., Graubard B.I., Troiano R.P., Berrigan D., Galuska D.A., Fulton J.E. (2022). Estimated number of deaths prevented through increased physical activity among US adults. JAMA Intern Med.

[10] Caspersen C.J., Powell K.E., Christenson G.M. (1985). Physical activity, exercise, and physical fitness: definitions and distinctions for health-related research. Public Health Rep.

[11] van Lummel R.C., Walgaard S., Pijnappels M., Elders P.J., Garcia-Aymerich J., van Dieën J.H. (2015). Physical performance and physical activity in older adults: associated but separate domains of physical function in old age. PLoS One.

[12] Dzik K.P., Kaczor J.J. (2019). Mechanisms of vitamin D on skeletal muscle function: oxidative stress, energy metabolism and anabolic state. Eur J Appl Physiol.

[13] Tieland M., Brouwer-Brolsma E.M., Nienaber-Rousseau C., van Loon L.J., De Groot L.C. (2013). Low vitamin D status is associated with reduced muscle mass and impaired physical performance in frail elderly people. Eur J Clin Nutr.

[14] Annweiler C., Henni S., Walrand S., Montero-Odasso M., Duque G., Duval G.T. (2017). Vitamin D and walking speed in older adults: systematic review and meta-analysis. Maturitas.

[15] Beaudart C., Buckinx F., Rabenda V., Gillain S., Cavalier E., Slomian J. (2014). The effects of vitamin D on skeletal muscle strength, muscle mass, and muscle power: a systematic review and meta-analysis of randomized controlled trials. J Clin Endocrinol Metab.

[16] Rosendahl-Riise H., Spielau U., Ranhoff A.H., Gudbrandsen O.A., Dierkes J. (2017). Vitamin D supplementation and its influence on muscle strength and mobility in community-dwelling older persons: a systematic review and meta-analysis. J Hum Nutr Diet.

[17] Muir S.W., Montero-Odasso M. (2011). Effect of vitamin D supplementation on muscle strength, gait and balance in older adults: a systematic review and meta-analysis. J Am Geriatr Soc.

[18] Bislev L.S., Grove-Laugesen D., Rejnmark L. (2021). Vitamin D and muscle health: a systematic review and meta-analysis of randomized placebo-controlled trials. J Bone Miner Res.

[19] Tabrizi R., Hallajzadeh J., Mirhosseini N., Lankarani K.B., Maharlouei N., Akbari M. (2019). The effects of vitamin D supplementation on muscle function among postmenopausal women: a systematic review and meta-analysis of randomized controlled trials. Excli J.

[20] Abshirini M., Mozaffari H., Kord-Varkaneh H., Omidian M., Kruger M.C. (2020). The effects of vitamin D supplementation on muscle strength and mobility in postmenopausal women: a systematic review and meta-analysis of randomised controlled trials. J Hum Nutr Diet.

[21] Bislev L.S., Wamberg L., Rolighed L., Grove-Laugesen D., Rejnmark L. (2022). Effect of daily vitamin D3 supplementation on muscle health: an individual participant meta-analysis. J Clin Endocrinol Metab.

[22] Bischoff-Ferrari H.A., Vellas B., Rizzoli R., Kressig R.W., da Silva J.A.P., Blauth M. (2020). Effect of vitamin D supplementation, omega-3 Fatty acid supplementation, or a strength-training exercise program on clinical outcomes in older adults: the DO-HEALTH randomized clinical trial. JAMA.

[23] Chou S.H., Cook N.R., Kotler G., Kim E., Copeland T., Lee I.M. (2024). Effects of supplemental vitamin D3, omega-3 fatty acids on physical performance measures in the VITamin D and OmegA-3 TriaL. J Clin Endocrinol Metab.

[24] Dupont J., Dedeyne L., Dalle S., Koppo K., Gielen E. (2019). The role of omega-3 in the prevention and treatment of sarcopenia. Aging Clin Exp Res.

[25] McGlory C., Calder P.C., Nunes E.A. (2019). The influence of omega-3 fatty acids on skeletal muscle protein turnover in health, disuse, and disease. Front Nutr.

[26] Huang Y.-H., Chiu W.-C., Hsu Y.-P., Lo Y.-L., Wang Y.-H. (2020). Effects of omega-3 fatty acids on muscle mass, muscle strength and muscle performance among the elderly: a meta-analysis. Nutrients.

[27] Graham Z.A., Lavin K.M., O’Bryan S.M., Thalacker-Mercer A.E., Buford T.W., Ford K.M. (2021). Mechanisms of exercise as a preventative measure to muscle wasting. Am J Physiol Cell Physiol.

[28] Flores-Bello C., Correa-Muñoz E., Sánchez-Rodríguez M.A., Mendoza-Núñez V.M. (2024). Effect of exercise programs on physical performance in community-dwelling older adults with and without frailty: systematic review and meta-analysis. Geriatrics (Basel).

[29] Gómez-Redondo P., Valenzuela P.L., Morales J.S., Ara I., Mañas A. (2024). Supervised versus unsupervised exercise for the improvement of physical function and well-being outcomes in older adults: a systematic review and meta-analysis of randomized controlled trials. Sports Med.

[30] Chou C.H., Hwang C.L., Wu Y.T. (2012). Effect of exercise on physical function, daily living activities, and quality of life in the frail older adults: a meta-analysis. Arch Phys Med Rehabil.

[31] Bischoff-Ferrari H.A., de Godoi Rezende Costa Molino C., Rival S., Vellas B., Rizzoli R., Kressig R.W. (2021). DO-HEALTH: Vitamin D3 - Omega-3 - Home exercise - Healthy aging and longevity trial - Design of a multinational clinical trial on Healthy aging among European seniors. Contemp Clin Trials.

[32] Middleton A., Fritz S.L., Lusardi M. (2015). Walking speed: the functional vital sign. J Aging Phys Act.

[33] Wolf A.M., Hunter D.J., Colditz G.A., Manson J.E., Stampfer M.J., Corsano K.A. (1994). Reproducibility and validity of a self-administered physical activity questionnaire. Int J Epidemiol.

[34] Ainsworth B.E., Haskell W.L., Herrmann S.D., Meckes N., Bassett D.R., Tudor-Locke C. (2011). 2011 Compendium of Physical Activities: a second update of codes and MET values. Med Sci Sports Exerc.

[35] Guralnik J.M., Simonsick E.M., Ferrucci L., Glynn R.J., Berkman L.F., Blazer D.G. (1994). A short physical performance battery assessing lower extremity function: association with self-reported disability and prediction of mortality and nursing home admission. J Gerontol.

[36] Gagesch M., Wieczorek M., Abderhalden L.A., Lang W., Freystaetter G., Armbrecht G. (2023). Grip strength cut-points from the Swiss DO-HEALTH population. Eur Rev Aging Phys Act.

[37] Wanigatunga A.A., Sternberg A.L., Blackford A.L., Cai Y., Mitchell C.M., Roth D.L. (2021). The effects of vitamin D supplementation on types of falls. J Am Geriatr Soc.

[38] Mazess R.B., Bischoff-Ferrari H.A., Dawson-Hughes B. (2021). Vitamin D: Bolus Is Bogus-A Narrative Review. JBMR Plus.

[39] Foroni M.Z., Cendoroglo M.S., Costa A.G., Marin-Mio R.V., do Prado Moreira P.F., Maeda S.S. (2022). FGF23 levels as a marker of physical performance and falls in community-dwelling very old individuals. Arch Endocrinol Metab.

[40] Elsurer Afsar R., Afsar B., Ikizler T.A. (2023). Fibroblast growth factor 23 and muscle wasting: a metabolic point of view. Kidney Int Rep.

[41] Bischoff-Ferrari H.A., Dawson-Hughes B., Staehelin H.B., Orav J.E., Stuck A.E., Theiler R. (2009). Fall prevention with supplemental and active forms of vitamin D: a meta-analysis of randomised controlled trials. BMJ.

[42] Bischoff-Ferrari H.A., Orav E.J., Abderhalden L., Dawson-Hughes B., Willett W.C. (2019). Vitamin D supplementation and musculoskeletal health. Lancet Diabetes Endocrinol.

[43] Bischoff-Ferrari H.A., Dawson-Hughes B., Platz A., Orav E.J., Stähelin H.B., Willett W.C. (2010). Effect of high-dosage cholecalciferol and extended physiotherapy on complications after hip fracture: a randomized controlled trial. Arch Intern Med.

