# Supplementary material for: Effects of vitamin D3, omega-3 fatty acids and a simple home exercise program on change in physical activity among generally healthy and active older adults: The 3-year DO-HEALTH trial
Source: J Nutr Health Aging. 2025 Mar 6;29(5):100528. doi: 10.1016/j.jnha.2025.100528 (PMC12180030; doi:10.1016/j.jnha.2025.100528)
Supplement: Supplementary file 1 [file mmc1.pdf]

SUPPLEMENTAL MATERIAL

Effects of vitamin D3, omega-3 fatty acids and a simple home exercise program on change in physical activity among generally healthy and active older adults: the 3-year DO-HEALTH trial

Kariem Hussein[1,2]*, Melanie Kistler-Fischbacher[1,2]*, Michèle Mattle[1,2], Caroline De Godoi Rezende Costa Molino[1,2], Li-Tang Tsai[1,2], Reto W. Kressig[3], E. John Orav[4], José A.P. da Sliva[5], Bruno Vellas[6], René Rizzoli[7], Gabriele Armbrecht[8], Egli Andreas[1,2], Bess Dawson-Hughes, Heike A. Bischoff-Ferrari[1,2]; DO-HEALTH Research group

*equal contributions

[1] Centre on Aging and Mobility, University of Zurich, Zurich, Switzerland

[2] Department of Geriatric Medicine and Aging Research, University of Zurich, Zurich, Switzerland

[3] University Department of Geriatric Medicine FELIX PLATTER and University of Basel, Basel, Switzerland

[4] Department of Biostatistics, Harvard T.H. Chan School of Public Health, Boston, MA, USA

[5] Centro Hospitalar e Universitário de Coimbra, Coimbra, Portugal; Centre for Innovation in Biomedicine and Biotechnology (CIBB), Faculty of Medicine, University of Coimbra, Coimbra, Portugal

[6] Gérontopôle de Toulouse, Institut du Vieillissement, Centre Hospitalo-Universitaire de Toulouse, Toulouse, France; UMR INSERM 1027, University of Toulouse III, Toulouse, France

[7] Division of Bone Diseases, Geneva University Hospitals and Faculty of Medicine, Geneva, Switzerland.

[8] Department of Radiology, Charité-Universitätsmedizin Berlin, Corporate Member of Freie Universität Berlin and Humboldt-Universität Zu Berlin, Berlin, Germany

[9] Jean Mayer USDA Human Nutrition Research Center on Aging, Tufts University, Boston, Massachusetts

Corresponding author
Bischoff-Ferrari Heike A., MD, DrPH
Dept. of Geriatric Medicine and Aging Research,
University of Zurich, Centre on Aging and Mobility, Tièchestrasse 99, 8037 Zürich, Switzerland
Email: heikea.bischoff-ferrari@uzh.ch

Supplemental Table 1. Use of vitamin D supplements in addition to the study medication at baseline and over the follow-up, overall and by treatment group.

	Overall	Vitamin D3	No vitamin D3	Omega-3	No Omega-3	SHEP	Control exercise
Baseline	522/2157 (24.2)	251/1076 (23.3)	271/1081 (25.1)	266/1073 (24.8)	256/1084 (23.6)	274/1081 (25.35)	248/1076 (23.1)
Year 1	544/1960 (27.8)	252/974 (25.9)	292/986 (29.6)	262/961 (27.3)	282/999 (28.2)	290/979 (29.62)	254/981 (25.9)
Year 2	553/1859 (29.8)	255/929 (27.5)	298/930 (32.0)	277/915 (30.3)	276/244 (29.2)	285/935 (30.48)	268/924 (29.0)
Year 3	627/1900 (33.0)	296/950 (31.2)	331/950 (34.8)	314/935 (33.6)	313/965 (32.4)	322/956 (33.68)	305/944 (32.3)
Abbreviations: SHEP, simple home exercise program							

Supplemental Table 2. P values for subgroup interactions [a], total PA and five times sit-to-stand

Subgroup	Vitamin D3	Omega-3s	SHEP
<u>**Total PA**</u>			
Sex (men, women)	0.59	0.36	0.28
Age (70 -74 yrs, ≥ 75 years)	0.61	0.20	0.87
<u>**STS**</u>			
Sex (men, women)	0.99	0.74	0.92
Age (70 -74 yrs, ≥ 75 years)	0.99	0.40	0.06

Abbreviations: STS, five times sit-to-stand; SHEP, simple home exercise program; PA, physical activity

[a] Interactions between the treatment groups and predefined subgroups were investigated in mixed effects models by adding the respective interaction terms. According to the main analysis, models were adjusted for age, linear spline at age 85, sex, BMI, prior fall, study site, time, and baseline measure of the outcome. For STS, a significant interaction between omega-3s and time was observed (P value=0.009), so the model included the interaction term for treatment*time.

Supplemental Table 3. P values for interactions between the 8 treatment group combinations and subgroups for gait speed and dominant grip strength

Subgroup	***P* value for all groups** [a]
<u>**Gait speed**</u>	
Sex (men, women)	0.99
Age (70 -74 yrs, ≥ 75 years)	0.51
<u>**Dominant grip strength**</u>	
Sex (men, women)	0.67
Age (70 -74 yrs, ≥ 75 years)	0.22

[a] Interactions between the treatment groups and predefined subgroups were investigated in mixed effects models by adding the respective interaction terms. No significant interaction between treatment groups and time was observed for either outcome, so the model omitted the interaction term for treatment*time. According to the main analysis, models were adjusted for age, linear spline at age 85, sex, BMI, prior fall, study site, time, and baseline measure of the outcome.

Supplemental Table 4. 3-year average change in STS and total PA for treatment combinations

Treatment group	Average across 3 yrs (95% CI) [a]	*P* value
**STS** [s]		
Vitamin D3 (vs. no vitamin D3)	-0.01 (-0.23, 0.20)	0.90
Omega-3s (vs. no omega-3s)	0.02 (-0.19, 0.24)	0.82
SHEP (vs. control exercise)	-0.14 (-0.36, 0.07)	0.19
Vitamin D3 + Omega-3s (vs. no vitamin D3, no omega-3s)	0.01 (-0.29, 0.32)	0.97
Vitamin D3 + SHEP (vs. no vitamin D3, control exercise)	-0.16 (-0.46, 0.14)	0.31
Omega-3s +SHEP (vs. no omega-3s, control exercise)	-0.12 (-0.42, 0.18)	0.44
Vitamin D3 + Omega-3s + SHEP (vs. no vitamin D3, no omega-3s, control exercise)	-0.13 (-0.50, 0.24)	0.49
**Total PA** [MET h/wk]		
Vitamin D3 (vs. no vitamin D3)	-7.1 (-12.7, -1.5)	0.01
Omega-3s (vs. no omega-3s)	-0.0 (-5.7, 5.6)	0.99
SHEP (vs. control exercise)	-2.0 (-7.6, 3.6)	0.48
Vitamin D3 + Omega-3s (vs. no vitamin D3, no omega-3s)	-7.2 (-15.2, 0.9)	0.08
Vitamin D3 + SHEP (vs. no vitamin D3, control exercise)	-9.1 (-17.1, -1.2)	0.02
Omega-3s +SHEP (vs. no omega-3s, control exercise)	-2.0 (-10.0, 5.9)	0.62
Vitamin D3 + Omega-3s + SHEP (vs. no vitamin D3, no omega-3s, control exercise)	-9.2 (-19.0, 0.6)	0.07

Abbreviations: STS, five times sit-to-stand; CI, confidence interval; MET, metabolic equivalent; PA, physical activity; SHEP, simple home exercise program; wk, week; yrs, years

[a] Estimates are from mixed effects models with change from baseline as the outcome. For STS, a significant interaction between omega-3s and time was observed (*P* value=0.009), so the model included the interaction term for treatment time. For PA, no significant interaction between treatment groups and time was observed, so the model omitted the interaction term for treatment*time. Additionally, the models controls for age, linear spline at age 85, sex, BMI, prior fall, study site, time, and corresponding baseline measure.

Supplemental Table 5. Quartiles of mean achieved 25(OH)D[a] levels across year 1, 2, and 3

Quartile of mean achieved 25(OH)D levels	N of participants at baseline	Mean (SD) [ng/mL]	Range (min-max) [ng/mL]
1	489	15.38 (3.88)	3.92 - 21.04
2	491	25.09 (2.29)	21.05 – 29.02
3	490	32.63 (2.24)	29.03 – 36.69
4	490	43.45 (5.79)	36.70 – 85.27

[a] Quartiles of achieved 25(OH)D levels were calculated based on the mean achieved 25(OH)D levels across years 1, 2, and 3.

Supplemental Table 6. Change from baseline in physical activity [MET h/wk] over 3 years by quartiles of achieved 25(OH)D levels across year 1, 2, and 3

Quartile	N participants at baseline	Unadjusted mean physical activity (95% CI), [MET h/wk]	Adjusted change from baseline in PA (95% CI) [a], [MET h/wk]	Difference compared to quartile 2 (95% CI)[a], [MET h/wk]	*P* value [a]
1	489	95.27 (87.75, 102.79)	-4.54 (-10.33, 1.26)	-7.20 (-15.37, 0.97)	0.08
2	491	106.96 (99.02, 114.90)	2.67 (-3.06, 8.39)	Reference	ref
3	490	104.37 (96.58, 112.16)	-7.26 (-12.91, -1.61)	-9.93 (-17.97, -1.89)	0.02
4	490	100.50 (93.09, 107.91)	-7.14 (-12.86, -1.43)	-9.81 (-17.93, -1.69)	0.02

Abbreviations: MET, metabolic equivalent
[a] Estimates are from a mixed effects model with change from baseline as the outcome. The model controls for age, linear spline at age 85, sex, prior fall, study site, BMI, time, baseline physical activity, and treatment effects of omega-3 and SHEP. The model did not include the treatment effect of vitamin D3, as the latter may be considered on the pathway of the association.
